# Studying a Flexible Polyurethane Elastomer with Improved Impact-Resistant Performance

**DOI:** 10.3390/polym11030467

**Published:** 2019-03-12

**Authors:** Jitang Fan, Ang Chen

**Affiliations:** State Key Laboratory of Explosion Science and Technology, Beijing Institute of Technology, Beijing 100081, China; 13269321551@163.com

**Keywords:** polyurethane elastomer, strain rate, impact-resistant performance, plastic zone, energy absorption

## Abstract

A flexible polyurethane elastomer (PUE) is studied, and the improved impact-resistant performance is revealed. Compressive stress–strain curves over a wide loading rate range were derived. Under static loading, the rubbery-like characteristics are demonstrated, which are flexible and hyperelastic, to process a large strain of about 60% followed by full recovery upon unloading. Under high-rate loadingcompared with the mechanical data of polyurethane elastomer (PUE) and polyurea (PUA) materials in the literature. Orderly parallel deformation bands were formed from carrying a large strain. The fibrils were found between deformation bands for enhancing the yield/plateau stress. A considerable plastic zone ahead of propagating crack with numerous crazes and microcracks was produced for realizing the dynamic strain energy absorption. This work presents a scientific innovation for developing outstanding impact-resistant polyurethane elastomers for transparent protection engineering.

## 1. Introduction

Nowadays, elastomers, a kind of soft, lightweight, and flexible polymer, have become one of the most frequently applied materials in dynamic events, due to their outstanding impact-resistant performance, such as large deformation, high shock absorption, low wave impedance, etc. [1,2]. In addition to the high specific strength (strength-to-weight ratio), they can play a key role in reducing the weight of the constructed protective structures while not abating the capability to resist impact loading or shock explosion, which will notably enhance the mobility of equipment and improve the safety and security.

Elastomer, with a material state between rubber and plastics, can not only be prepared and processed as a plastic but also has rubber-like mechanical performance [3]. They are viscoelastic/hyperelastic, responding to static loading and becoming stiff when subjected to an impact loading, which reflects the phenomena of high strain rate dependency [4,5,6]. Among the elastomers, polyurea (PUA) and polyurethane elastomer (PUE) are the most widely investigated and applied in protective engineering [1,5]. PUA is an elastomeric material made by isocyanate and amine [7]. By using spray technology, PUA can be coated on the surface of various substrates, which can extensively strengthen the mechanical properties against shock loading [8,9]. PUE was developed to substitute the traditional rubbers [10], which has the performance of high elasticity, flexibility, and resistance to impact. The preparation technology and physical properties have been reported in [11]. It can be prepared through a chemical reaction between polyols and isocyanates [12]. From a molecular perspective, the oligomer polyols constitute soft segments, while the isocyanates and chain extenders constitute hard segments [13]. The nature of interaction between the soft segments and hard segments endows the material performance transition from a rubbery-like behavior at low strain rates to a glassy-like behavior at high strain rates [14,15], which contributes to the difference of mechanical properties and their rate of dependency.

As a soft and flexible material, elastomer usually has a large deformation capability because its glass transition temperature ($T_{g}$) is lower than room temperature. This physical feature also produces the potential of a high dependency of mechanical properties on loading rate [14,15,16]. Thus, under high-rate loading, the characteristics of the stress–strain curve show an initially linearly elastic deformation to yielding, followed by large straining at a plateau stress until the final failure or unloading [14,16]. In the family of elastomer materials, plateau stress is a key property in determining the dynamic strain energy absorption, i.e., the area under the stress–strain curve, which can evaluate the impact-resistant performance. Thus, through material design, enhancing the plateau stress of elastomers under dynamic loading can effectively improve the impact resistance.

In this study, through composite design of the soft segments and hard segments, a novel polyurethane elastomer (PUE) was developed, which is transparent, soft, and flexible under static loading at room temperature. A modified split Hopkinson pressure bar (SHPB) device was used to characterize how the dynamic compressive response and the stress–strain curves at various strain rates wwere derived [16]. By analyzing the mechanical data and comparing it with the reported elastomer materials, higher plateau stress and comparable strain are achieved, which indicates an improved impact-resistant capability. Then, deformation and fracture behavior under dynamic loading were observed by scanning electron microscope (SEM) for illustrating the intrinsic physical mechanisms. This study, by composite material design, gives a high-performance polyurethane elastomer with improved impact resistance for enhancing the security of the transparent protection engineering.

## 2. Experimental Procedure

### 2.1. Material Preparation and Molecular Structure

Polyurethane elastomer (a group of segmented copolymers) is a kind of soft polymer with linear molecular chain structure [17]. The main molecular chains are formed by the urethane groups attached to the aliphatic or aromatic structures with a random distribution [18,19]. The structure of the aliphatic, aromatic, and/or ester ring group is in an isocyanate monomer. The hard segments (HS) are composed of isocyanates and chain extenders, which form stiff urethane units, and soft segments (SS) are based on aliphatic polyethers, polyesters, or polydimethylsiloxanes, named as macrodiols [20,21,22,23].

**Figure 1:**
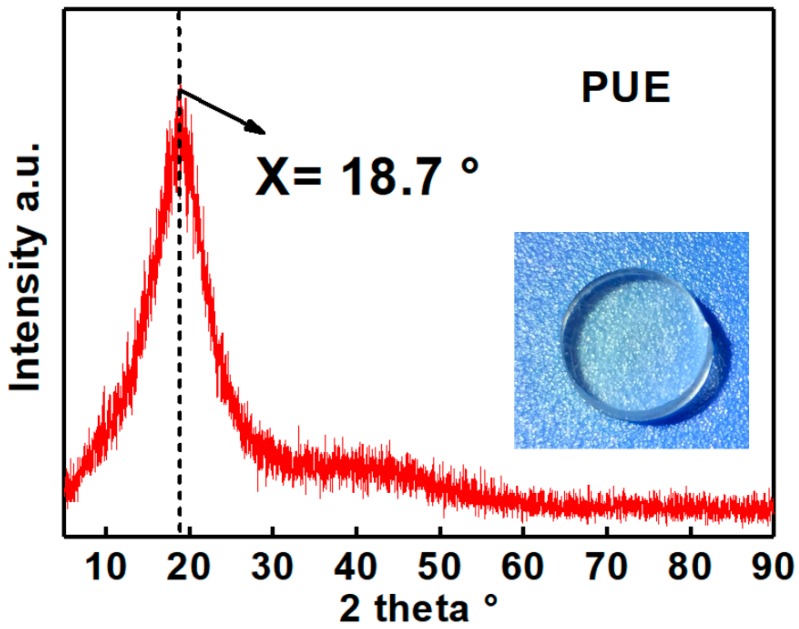
XRD analysis of the developed polyurethane elastomer (PUE) material, revealing a fully amorphous molecular microstructure, and the transparent characteristic as shown in inset.

In this study, two kinds of transparent prepolymer components of oligomer polyol, small molecule chain extender, catalyst, and isocyanate are directly mixed to develop the novel polyurethane elastomer. These prepolymer components are commercial products of SFP supramolecular. The synthesized material is a kind of PUE, which is a family member of elastomers. The developed PUE material is transparent, as seen in the inset of Figure 1. X-ray diffraction (XRD), a non-destructive analytical technique for characterizing the atomic and molecular structure of materials, was employed to analyze the PUE material. The XRD pattern consists of a broad diffraction peak without any detectable sharp peaks, which indicates the amorphous molecular structure. This microstructural feature is in line with the transparent characteristic of the PUE material. Herein, the broad diffraction peak appears at an angle of about 20°, which is close to the observation in other polyurethane materials [24,25,26,27,28,29,30].

### 2.2. Quasi-Static Compression Tests and Properties

Quasi-static compressive tests were carried out using a universal MTS testing machine at room temperature [29]. Lubrication was performed to reduce the contact friction between the specimen and loading platens. The tested specimens are cylindrical with a diameter of 6 mm and length of 5 mm, and the strain rate was set as 0.001/s. Tests were conducted three times to confirm the repeatability of experimental results [30]. During each test, a camera was employed to record the deformation process of the specimen.

**Figure 2:**
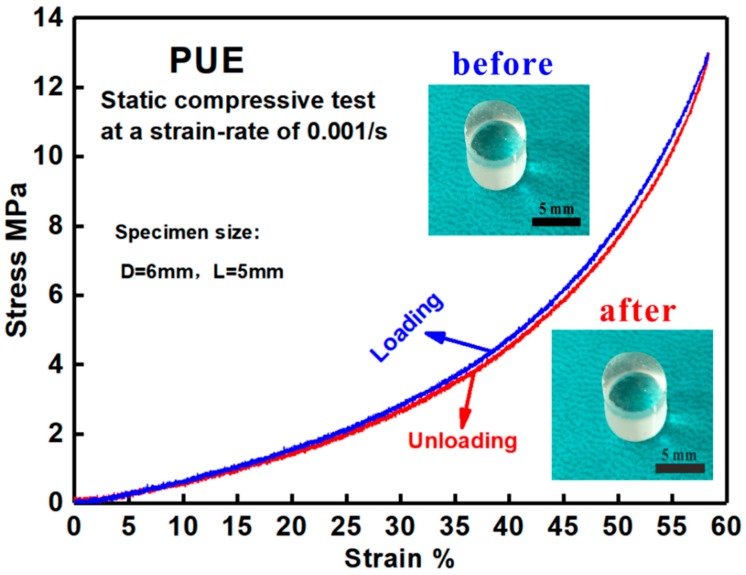
Quasi-static compressive stress–strain curves of the developed PUE material with a loading–unloading round and the fully recoverable deformation characteristics.

Figure 2 shows the quasi-static compressive stress–strain curves of the developed PUE material, with a loading–unloading cycle. In the loading process, it displays a nonlinear hyperelastic deformation until 55% strain, without observable yielding behavior. In the unloading process, the stress-strain curve has almost the same path with the loading part, which indicates a limited hysteresis loop [16]. This phenomenon implies a slight, even no change, in the orientation or waviness of individual molecular chains upon static loading. Thus, the fully recoverable deformation characteristic is provided, which is also confirmed by comparing the geometrical dimensions of the specimens before and after loadings (see the insets in Figure 2). Thus, the recoverability results in the disappearance of permanent deformation [31]. Hence, the traditional definition of yield stress as the stress at a strain of 0.2% is not suitable. Herein, the yield stress is considered as 0 MPa for PUE material under static loading.

### 2.3. Dynamic Compression Tests at Various Strain Rates

**Figure 3:**
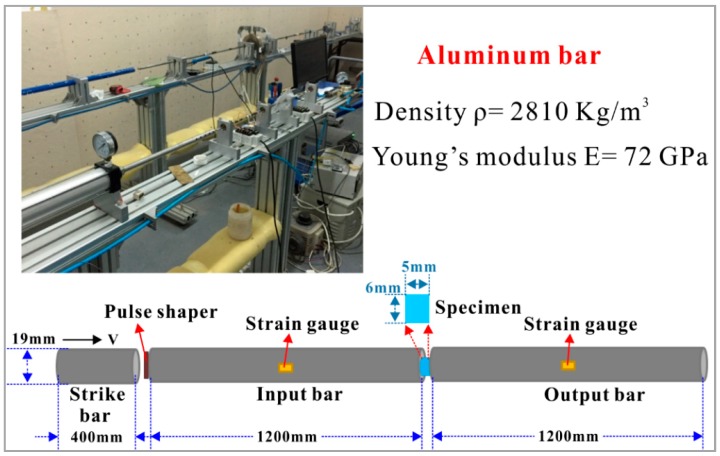
Physical image, schematic diagram, and detailed specifications of the employed split Hopkinson pressure bar (SHPB) device with aluminum bars.

Split Hopkinson pressure bar (SHPB) apparatus, mainly consisted of strike bar, input bar, and output bar, was employed to perform the dynamic compression tests of the developed polyurethane elastomer (PUE) [32]. The physical image, schematic diagram, and detailed specifications of the SHPB device with aluminum bars are shown in Figure 3. The diameter, mass density, and Young’s modulus of the aluminum bars are 19 mm, 2810 kg/m^3^, and 72 GPa, respectively.

The specimen size in SHPB tests plays an essential role in obtaining reliable experimental results. The ideal length-to-diameter ratio (L/D) of the tested polymer specimen is 0.5–1. The cross section of the deformed specimen should be within the pressure bar. Lubrication was performed between the specimen and bars, which is important for minimizing the effect of friction and achieving dynamic stress equilibrium in the loaded specimen [16,33,34,35]. During an SHPB test, the change in specimen size with timing cannot be precisely measured. The initial cross-sectional area and length of specimen are used to calculate the dynamic stress, strain, and strain rate, which are in the engineering version [6,36]. The detailed working principle has been reported in [37,38,39,40,41,42,43]. One-dimensional wave propagation theory is assumed to derive the stress, strain, and strain rate with timing, based on the recorded strains of the input and output bars [17]. They are expressed as seen below:

(1) $$\sigma_{s}\left(t\right)=E_{0}\frac{A_{0}}{A_{s0}}\varepsilon_{t}\left(t\right),$$

(2) $$\varepsilon_{s}\left(t\right)=-\frac{2C_{0}}{L_{s0}}\int_{0}^{t}\varepsilon_{r}\left(t\right)dt,$$

(3) $${\dot{\varepsilon }}_{s}\left(t\right)=-\frac{2C_{0}}{L_{s0}}\varepsilon_{r}\left(t\right),$$

where $\sigma_{s}\left(t\right)$, $\varepsilon_{s}\left(t\right)$, and ${\dot{\varepsilon }}_{s}\left(t\right)$ are the stress, strain, and strain rate of the tested specimen; $\varepsilon_{r}\left(t\right)$ and $\varepsilon_{t}\left(t\right)$ are the recorded strains of the input bar and output bar, respectively; $A_{s0}$ and $L_{s0}$ are the initial cross-sectional area and length of the tested specimen; $E_{0}$ is the Young’s modulus of bars; $A_{0}$ is the cross-sectional area of bars; and $C_{0}=\sqrt{E_{0}/\rho_{0}}$ ($\rho_{0}$ is the density of bars) is the wave velocity in bars.

## 3. Dynamic Experimental Results and Discussions

### 3.1. Dynamic Stress–Strain Characteristics

**Figure 4:**
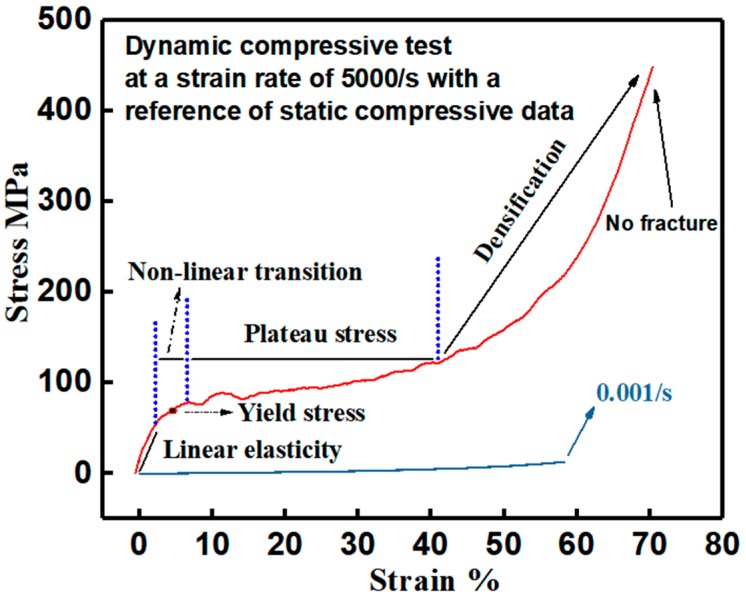
Representative engineering stress–strain curve of the developed PUE material at a strain rate of 5000/s with a reference curve showing the static compressive data at a strain rate of 0.001/s.

Dynamic compressive tests were conducted on the developed PUE material via an aluminum SHPB device. To illustrate the dynamic stress–strain characteristics, the representative engineering stress–strain curve at a strain rate of 5000/s was derived, as shown in Figure 4. The initial deformation is linearly elastic with a slope of about 1300 MPa, named as dynamic Young’s modulus. Afterwards, it is a nonlinear transition at an intermediate stress of about 58 MPa, determined as dynamic yield stress. Then, a large strain is processed under plateau stress until densification accompanied with notable stress rise [44]. In this process, no obvious softening occurs. Thus, dynamic yield stress is consistent with the plateau stress, which is a key parameter to evaluate the strain energy absorption and impact-resistant performance of the PUE material. In addition, in contrast with the hyperelastic performance under static loading at a strain rate of 0.001/s, dynamic stress–strain curve shows the evident linearly elastic deformation and yielding behavior. It is a typical characteristic of rubbery-like behavior at a low strain rate and processes a transition into glassy-like behavior at a high strain rate. This transition is caused by the increase of strain rate [45,46,47,48], which contributes to the dynamic hardenability of the flexible PUE material and the advance in impact resistance.

Therefore, the developed PUE is a hyperelastic material under static loading, while being an elastoplastic material under dynamic loading, which is classified as the plastic material [49,50]. The physical reason is that when subjected to a high-speed impact, the glass transition temperature ($T_{g}$) of the PUE material will rise above the ambient temperature, i.e., room temperature in the current case, which is caused by the high frequency loading and high pressure [51]. When the transient glass transition is processed, the transition in mechanical behavior is resulted.

### 3.2. Strain Rate Dependency and the Improved Impact-Resistant Performance

**Figure 5:**
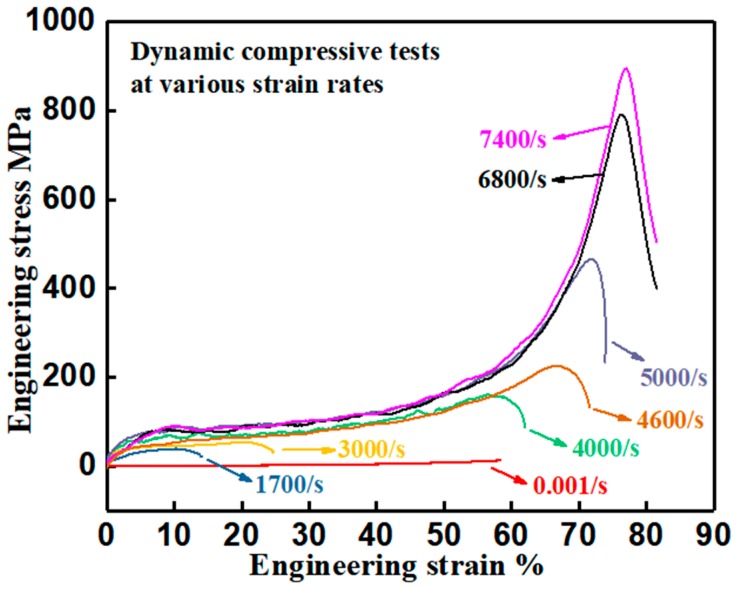
Representative dynamic compressive engineering stress–strain curves of the developed PUE material at various strain rates.

Strain rate dependency of the mechanical properties of the developed PUE material was studied at room temperature within the strain rate range of 0.001/s to 7400/s. Through comparison, the improved impact-resistant capability was derived. Figure 5 displays the representative engineering stress–strain curves at different strain rate levels (derived from the first loading pulse in each SHPB test) [16]. They show an obvious difference in yield stress and plateau stress, which belongs to the domain of strain rate dependency. Under impact loading, due to the internal/interior friction effect of the molecular chains, the activation energy of segment movement will increase with the increase of loading rate, which leads to the enhancement of mechanical strength [52].

**Figure 6:**
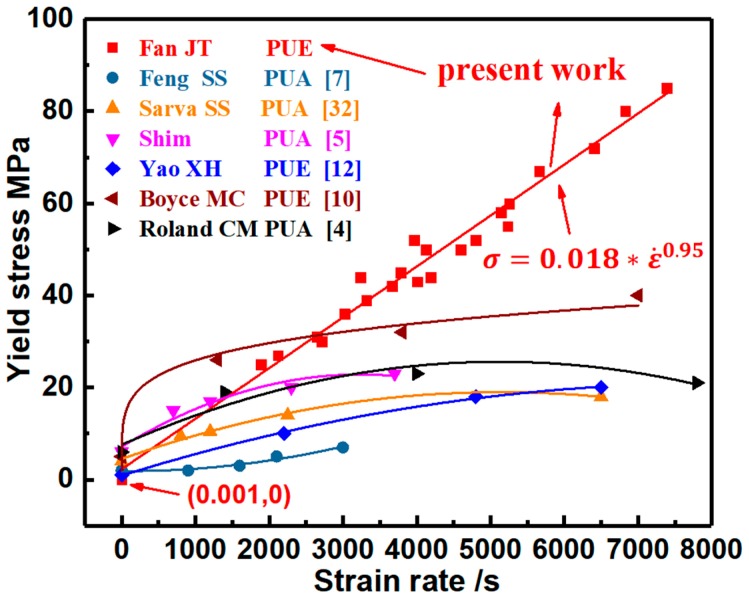
Improvement in the dynamic yield stress of the developed PUE material is at a higher strain rate as compared with the reported elastomers of polyurea (PUA) and polyurethane elastomer (PUE), indicating an enhancement in impact resistance.

Yield stress, instead of plateau stress, is investigated with the increase of strain rate for quantitatively illustrating the strain rate-dependent mechanical properties and the improved impact-resistant performance, as shown in Figure 6. The notable increase of yield stress upon an increasing loading rate is indicated. A power law function was employed to characterize the strain rate dependency of yield stress [53], which can be numerically expressed as $\sigma =0.018*\;{\dot{\varepsilon }}^{0.95}$ by fitting the experimental data. Based on these experimental data, the pronounced increase of yield stress with the increase of strain rate was achieved in the developed PUE material because of the high strain rate dependency. Thus, dynamic hardening capability is attained. Compared with the other reported polyurea (PUA) and polyurethane elastomer (PUE), the developed PUE material has a higher dynamic yield stress (see Figure 6) and performs a comparable strain, which indicate the improvements of strain energy absorption and impact-resistant capability. Herein, through comparison in the family of elastomer materials, dynamic mechanical properties of the developed PUE material are higher at a high strain rate, which is the advance achieved in this work. The deformation and fracture mechanisms will be analyzed to clarify the intrinsic physical reasons.

Eyring’s equation can be used to illustrate the strength of polymer materials related to the applied strain rate and inherent material parameters [54,55], which is expressed as seen below:

(4) $$\frac{\sigma_{y}}{T}=\frac{\Delta U}{vT}+\frac{R}{v}\ln\left[\frac{2\dot{\varepsilon }}{e_{0}}\right],$$

where $\sigma_{y}$ is the yield stress; T is the ambient temperature; ΔU is the activation energy of plastic deformation/flow, i.e., the height of the potential energy barrier of two adjacent equilibrium positions for element units to jump; v is the activation volume of element motion unit; $\dot{\varepsilon }$ is the strain rate; $e_{0}$ is the pre-exponential factor; and R is the gas constant. Under dynamic loading at a given strain rate, yield stress is correlated with the material parameters of ΔU and v. In the developed PUE material, the hard segments can enhance the height of the potential energy barrier of two adjacent equilibrium positions for element units to jump, which contribute to the improvement of activation energy, ΔU. The soft segments can reduce the activation volume of element motion unit, v, and produce the deformation strain. Thus, by composite design of the hard and soft segments, the enhancement in dynamic yield stress with considerable deformation strain (see Figure 5 and Figure 6) is achieved, which leads to the improvement of strain energy absorption and impact resistance.

### 3.3. Mechanisms of Dynamic Yielding and Straining

**Figure 7:**
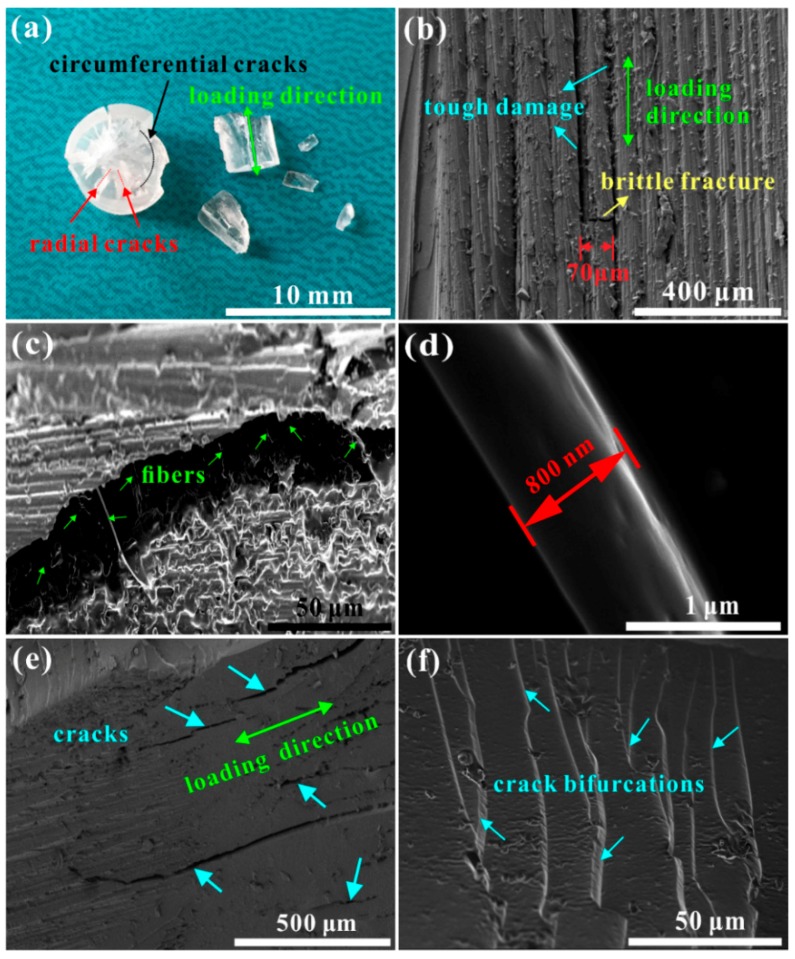
Post-test observations on the deformation and fracture behavior for revealing the mechanisms of dynamic yielding and straining of the developed PUE material: (**a**) cracking pattern and fracture mode; (**b**) uniform deformation bands elongated in the loading direction with two kinds of cracks; (**c**) fine fibrils linking an opening crack localizing between the deformation bands; (**d**) 800 nm diameter of a fibril; (**e**) numerous cracks formed after a considerable straining; and (**f**) brittle cracking behavior to fracture deformation bands, resulting in the final dynamic failure.

Mechanisms of dynamic yielding and straining of the developed PUE material were investigated by the post-test observations on deformation and fracture behavior, as shown in Figure 7. Figure 7a shows the cracking pattern and fracture mode. Two directions of the radial and circumferential cracks are formed (marked by the arrows with different colors), and the final fracture is along the loading direction caused by these two directional cracks. This phenomenon is due to the localized heterogeneous stress distribution inside the cylindrical specimen under SHPB loading [56]. Along the loading direction, numerous deformation bands are formed and elongated, which construct a uniform parallel pattern (see Figure 7b). These deformation bands are expected to be induced by lateral tensile stress upon an applied dynamic compressive loading [16,55], which carry the considerable strain. By measurement, they have an average width of about 70 µm. Two kinds of cracks are found. One is along the deformation bands and localizes between them, which contributes to the tough damage. The other is vertical to the deformation bands and cuts through them, which induces the brittle fracture.

For the tough damage, fine fibrils are observed, which link an opening crack localizing between the deformation bands (see Figure 7c). A zoomed-in observation shows that these fibrils are extended and become slim with a diameter of 800 nm (see Figure 7d). Also, these fibrils tangle the elongation of deformation bands, which will contribute to the high plateau stress during straining (see Figure 5 and Figure 6). Accompanying the deformation, more cracks initiate and propagate along the loading direction and some of the slimmed deformation bands are broken, which induces the interlinking of these cracks (see Figure 7e). By interlinking the parallel cracks, deformation bands are cut through, which forms the vertical cracks and brittle fracture. Figure 7f shows the typical brittle fracture characteristics of numerous crack bifurcations, indicating an unstable crack propagation to induce the final failure under dynamic loading.

### 3.4. Mechanisms of Strain Energy Absorption and Impact Resistance

**Figure 8:**
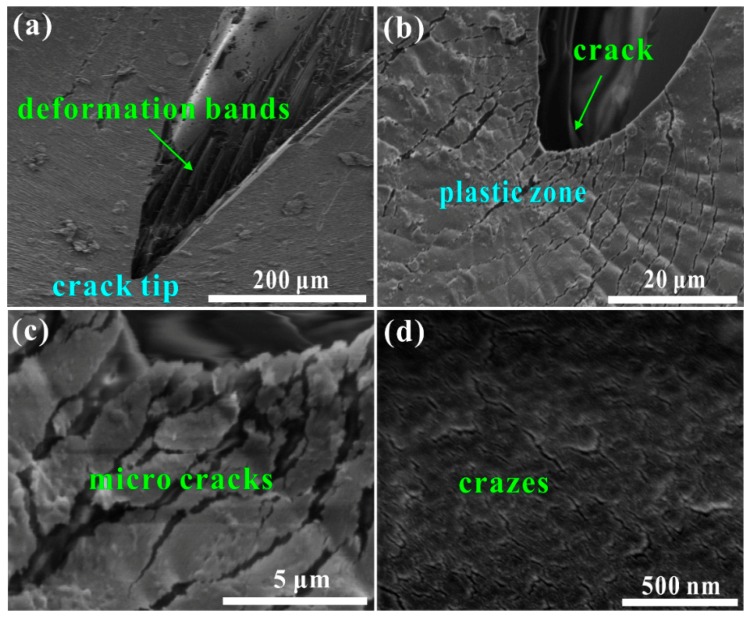
Post-test observations on the cracking behavior for revealing the mechanisms of strain energy absorption and impact resistance of the developed PUE material: (**a**) a growth crack with numerous parallel deformation bands inside the opening mouth; (**b**) a large plastic zone ahead of crack; and (**c**,**d**) high-density microcracks and crazes in the plastic zone.

Post-test observations using SEM were conducted for revealing the cracking behavior and the inherent mechanisms of dynamic strain energy absorption and impact-resistant capability of the developed PUE material (see Figure 8). Figure 8a shows a growth crack in the deformed specimen, which has numerous parallel deformation bands inside the opening mouth. They correspond with the observations on fracture surface as shown in Figure 7b, which indicates a large deformation. Ahead of the crack, a large plastic zone is formed, which blunts the crack and prevents its propagation (see Figure 8b) [29]. In the plastic zone, numerous examples of dynamic damage are found, which are indicated by the high-density microcracks and crazes (see Figure 8c,d). The spacing between microcracks is at submicron level and between crazes is at nanometer level, which demonstrates the high-density dynamic damage. Such the damage is premature without inducing fracture but can effectively suppress the crack propagation and growth. Thus, the resultant dynamic strain energy absorption is considerable, which contributes to the improvement of impact-resistant performance, as illustrated in Figure 6.

## 4. Conclusions

This work conducted a series of studies on the mechanical behavior of a developed PUE material in the strain rate range of 0.001/s–7400/s at room temperature. The physical mechanisms of the improved impact resistance are clarified. This is significant in providing a scientific route for the development of outstanding impact-resistant flexible polymers for designing high-performance transparent protection structures. Concluding remarks are as follows.

Quasi-static compressive tests with loading–unloading cycles reveal that the developed PUE material displays a hyperelastic deformation behavior under large strain with full recovery upon unloading, and is soft, flexible, and rubber-like.

High-rate compressive tests reveal that the developed PUE material displays an initially linear elasticity to yielding, followed by considerable straining at high plateau stress until densification to the final unloading or fracture, which is stiff, elastoplastic and glassy-like.

Strain rate dependency of mechanical properties is formulated by a power law relation, which indicates a fast increase of yield/plateau stress with the increase of strain rate. The higher dynamic yield/plateau stress and the comparable large strain are produced, as compared with the dynamic mechanical data of the reported polyurethane elastomer (PUE) and polyurea (PUA) materials. Thus, dynamic strain energy absorption and impact-resistant capability are improved.

The mechanism of carrying the large strain is the formation of the orderly parallel deformation bands along the compressive stress axis. The mechanism of enhancing the yield/plateau stress with the increase of strain rate is the formation and elongation of the fibrils between deformation bands. The mechanism of improving the dynamic strain energy absorption and impact-resistant capability is the impediment of the propagating crack by producing considerable plastic zone, which is full of high-density crazes and microcracks.
